# The Power of Molecular Dynamics Simulations and Their Applications to Discover Cysteine Protease Inhibitors

**DOI:** 10.2174/1389557523666230901152257

**Published:** 2023-09-27

**Authors:** Igor José dos Santos Nascimento, Joilly Nilce Santana Gomes, Jéssika de Oliveira Viana, Yvnni Maria Sales de Medeiros e Silva, Euzébio Guimarães Barbosa, Ricardo Olimpio de Moura

**Affiliations:** 1 Department of Pharmacy, Cesmac University Center, Maceió, 57051-160, Brazil;; 2 Department of Pharmacy, Drug Development and Synthesis Laboratory, State University of Paraíba, Campina Grande, 58429-500, Brazil;; 3 Post-graduate Program in Pharmaceutical Sciences, State University of Paraíba, Campina Grande, 58429-500, Brazil;; 4 Post-graduate Program in Bioinformatics, Bioinformatics Multidisciplinary Environment, Federal University of Rio Grande do Norte, Natal, Brazil;; 5 Post-graduate Program in Pharmaceutical Sciences, Faculty of Pharmacy, Federal University of Rio Grande do Norte, Natal, Brazil

**Keywords:** Neglected tropical diseases, molecular modeling, computer-aided drug design, computational chemistry, cysteine protease, molecular mechanics

## Abstract

A large family of enzymes with the function of hydrolyzing peptide bonds, called peptidases or cysteine proteases (CPs), are divided into three categories according to the peptide chain involved. CPs catalyze the hydrolysis of amide, ester, thiol ester, and thioester peptide bonds. They can be divided into several groups, such as papain-like (CA), viral chymotrypsin-like CPs (CB), papain-like endopeptidases of RNA viruses (CC), legumain-type caspases (CD), and showing active residues of His, Glu/Asp, Gln, Cys (CE). The catalytic mechanism of CPs is the essential cysteine residue present in the active site. These mechanisms are often studied through computational methods that provide new information about the catalytic mechanism and identify inhibitors. The role of computational methods during drug design and development stages is increasing. Methods in Computer-Aided Drug Design (CADD) accelerate the discovery process, increase the chances of selecting more promising molecules for experimental studies, and can identify critical mechanisms involved in the pathophysiology and molecular pathways of action. Molecular dynamics (MD) simulations are essential in any drug discovery program due to their high capacity for simulating a physiological environment capable of unveiling significant inhibition mechanisms of new compounds against target proteins, especially CPs. Here, a brief approach will be shown on MD simulations and how the studies were applied to identify inhibitors or critical information against cysteine protease from several microorganisms, such as *Trypanosoma cruzi* (cruzain), *Trypanosoma brucei* (rhodesain), *Plasmodium spp.* (falcipain), and SARS-CoV-2 (M^pro^). We hope the readers will gain new insights and use our study as a guide for potential compound identifications using MD simulations.

## 1 INTRODUCTION

The large family of enzymes with the function of hydrolyzing peptide bonds, called peptidases or cysteine proteases (CPs), are divided into three categories according to the peptide chain involved: *i)* endopeptidases (bromelain, ficain, papain, and cathepsins), and, *ii)* exopeptidases (carboxypeptidase B, and cathepsin X) [1, 2]. Regarding the catalytic mechanism, exopeptidases promote cleavage close to the C- or N-terminus of substrates, while endopeptidases promote cleavage in distant regions of the C- or N-terminus [2]. Furthermore, they can be categorized based on the reactive group involved in catalysis: serine, cysteine, aspartic endopeptidases, and metalloendopeptidases [1]. Cysteine carboxypeptidases are exopeptidases that cleave polypeptides at the C-terminus [1]. They are proteases with a reactive thiol, one of the oldest in the literature, present in several microorganisms, including viruses, bacteria, protozoa, plants, and others, constantly explored as main targets in medicinal chemistry and drug development work [3-7].

CPs have an average molecular mass between 21-30 kDa and have the function of catalyzing the hydrolysis of peptide bonds of amide, ester, thiol ester, and thioester and can be divided into five clans: *i)* papain-like (CA); *ii)* viral chymotrypsin-like CPs (CB); *iii)* papain-like endopeptidases of RNA viruses (CC); *iv)* legumain-type caspases (CD); and *v)* showing active residues of His, Glu/Asp, Gln, Cys (CE) [2, 8]. Most CPs are evolutionarily related to papain and show a common fold. A central feature is that activation requires proteolytic cleavage of the N-terminal pro-region, also functioning as an enzyme inhibitor [2]. In drug design studies, it is essential to know the CP category and start the design process based on the catalytic mechanism [9, 10].

**Fig. (1):**
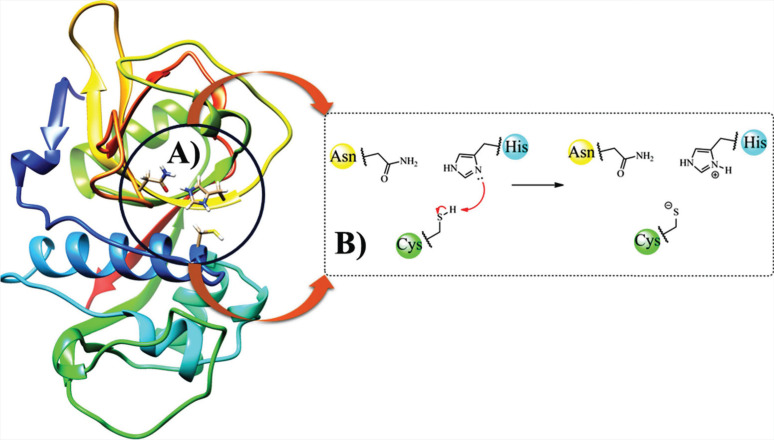
General catalytic mechanism of cysteine proteases using cruzain (PDB id: 1AIM): ***A)*** active site and ***B)*** mechanism.

The basis of the catalytic mechanism of CPs is the essential cysteine residue present in the active site, with the function of hydrolysis of peptide bonds [11, 12]. The thiol reactivity is commonly increased due to the proximity of a histidine residue that acts as a base, in which the sulfhydryl group (SH) and imidazole form a thiolate-imidazolium catalytic dyad (Fig. **1**). Often, this histidine load is stabilized by a highly conserved proximal asparagine residue. Furthermore, glutamine residues commonly form the oxyanion hole, which is crucial for producing an electrophilic center and stabilizing the tetrahedral intermediate in hydrolysis [11, 12]. Interestingly, the thiolate-imidazolium ionized groups allow for a wide pH range for optimal enzyme activity, with pKa 4 for cysteine and 8.5 for histidine. Further, other active site residues function as charge stabilizers. Hydrolysis mechanisms are well elucidated, in which the enzyme binds to unstable tetrahedral intermediates before returning to its active state [12]. Often, these mechanisms are studied through computational methods that aid in discovering new information about the catalytic mechanism and identifying possible inhibitors [13-15].

The role of computational methods during drug design and development stages is increasingly prominent [16]. Methods in Computer-Aided Drug Design (CADD) accelerate the discovery process, increase the chances of selecting more promising molecules for experimental studies, and can identify critical mechanisms involved in the pathophysiology and molecular pathways of action [16-18]. Among these methods, Molecular dynamics (MD) simulations are essential in any drug discovery program due to their high capacity for simulating a physiological environment capable of unveiling significant inhibition mechanisms of new compounds against target proteins, especially CPs [19]. It is a physics-based computational simulation method that studies the molecular motions of atoms and molecules using Newton's equations of motion [20]. In this way, it is possible to understand mechanistic events that occur at a macromolecular scale and unlock essential information that can be used in the hit-to-led drug discovery process [20-22].

Due to the importance of cysteine proteases for maintaining the normal physiology of several microorganisms, their promising potential in drug discovery studies, and the versatility of MD simulations, current trends in drug discovery against CPs using simulations of MD will be presented here. MD. Here, a brief approach will be shown on MD simulations and how the studies were applied to identify inhibitors or critical information against *Trypanosoma cruzi* (Cruzain), *Trypanosoma brucei* (Rhodesain), *Plasmodium* spp. (Falcipain), and SARS-CoV-2 (M^pro^). We hope the readers will gain new insights and use our study as a guide for potential compound identifications using MD simulations.

## 2 MOLECULAR DYNAMICS SIMULATIONS

The study of macromolecular structures and their biological functions has been critical in understanding their interactions and bodily functions [23]. Implementing X-ray crystallography and Nuclear Magnetic Resonance (NMR) techniques originate 3D databases of macromolecule information [23, 24]. These structures' study and prediction have supported the understanding of biological events through computational methodologies. Among these tools, Structure-Based Drug Design (SBDD) has been the basis for using 3D macromolecule structures by application in molecular docking, molecular modeling, and molecular dynamics simulations [25].

To provide flexibility and solve several problems related to molecular docking, theoretical techniques, such as molecular dynamics (MD) simulations, emerge as a great approach to obtaining an image of macromolecular dynamic properties. In this process, several conformations are generated through the movement of particles as a function of time [20, 26]. For example, there are studies involving proteins in solution [27], protein-ligand complex [28], membrane-embedded proteins [29], or large macromolecular complexes such as DNA [30]. The following sections will highlight new studies and issues to understand the theoretical foundations, computational resources, software performance, and applications in drug design and discovery.

### 2.1 Theoretical Fundamentals

A molecular system, such as proteins of interest, is represented in atoms as point masses. Given the positions of all the atoms in the system (*e.g.*, a protein surrounded by water) can calculate the force exerted on each atom [31]. This system has a random initial velocity influenced by a classical force field from molecular mechanics (MM). This force field fits with quantum mechanical (QM) calculations. Then there is a time propagation in femtoseconds, integrating Newton's equations of motion in small time steps. Once the system is built, force fields obtain the forces acting on all atoms, deriving these equations [32, 33]. They are called force fields because they describe the contributions of various atomic forces that govern molecular dynamics. Using complex equations, they are estimated from an interaction equation between chemically bonded and non-bonded atoms [33]. In this way, connections' length, angles, and rotation are modeled using simple springs and dihedral angles. In this way, unbound forms arise through van der Waals interactions, using the Lennard-Jones potential [34], and charged interactions (electrostatic) using Coulomb's law [35].

Among the existing and commonly used force fields in MD simulations are AMBER [36], CHARMM [37], and GROMOS [38], differing in their parameterization but providing similar results. These parameters are not interchangeable, and not all existing force fields can represent all types of molecules. For example, CHARMM has optimized and validated parameters for proteins, lipids, and drug-like ligands [37]. On the other hand, AMBER is commonly used for proteins, DNA, RNA, carbohydrate, lipid, ligands, and ions [36], while GROMOS is compatible with mono-, di-, oligo-, or polysaccharides [38, 39].

After calculating the forces on each of the atoms in the system, the other configurations are done manually. Thus, the objective is to create a box and insert molecules representing a biological system of interest. This process implies correcting structural errors, ionizing amino acids, adding counter-ions and solvents, minimizing energy, and applying system equilibrium at the desired temperature and pressure [31]. All parameters are chosen to simulate the right environment for the targeted analysis. After creating the box containing the balanced system, this propagation moves the system forward in time. In it, forces are used by differentiating the potential of the force field on the interactions between atoms, new velocities, and positions for all particles. This results in a molecular system trajectory in a 3D movie describing its evolution over time and the set of conformations [40].

The MD simulation interpretability arises through the generation of statistical analysis, visualized in graphs, which indicate the observed deviations of the system. The Root-Mean-Square Deviation (RMSD) is responsible for identifying, frame by frame, the deviation variation. Thus, it is possible to observe through the plateau if the system stabilizes. In this way, the RMSD can be calculated for any molecule in the system, such as macromolecules or ligands [41]. On the other hand, the Root-Mean-Square Fluctuation (RMSF) corresponds to an RMSD over time, identifying which residues are more spatially mobile. In this context, it is possible to determine which regions within the macromolecule have the greatest and least deviation [42].

Furthermore, the most reported in the literature, other evaluations can be observed. The Radius of Gyration (R_g_) can evaluate the folding of peptides and proteins, identifying the displacement of the mass center concerning an axis. The bulkier the protein, the greater it's R_g_ [43]. On the other hand, the hydrogen bond (H-bond) plots to calculate and analyze hydrogen bonds are determined based on the angle and distance between hydrogen, donor, and acceptor. The -OH and -NH groups are considered donors, while the -O and -N are acceptors by default [44].

### 2.2 Activity Determination

MD simulations can add flexibility to the ligand and a biological target and estimate binding affinity. Linear interaction energy (LIE) [45], Molecular Mechanics Poisson–Boltzmann Surface Area (MM-PBSA) [46, 47], and alchemical perturbation (AP) [48] are examples of methods that can be employed to estimate free energy dissociation constants or other related affinity measures. The first method is simpler and considers only End Points, the bound and unbound state, to estimate affinity. The last two methods consider statistical mechanics with more rigorous or accelerated sampling, being, however, more computationally expensive [48]. The first of these methods is the linear interaction energy (LIE) [45], which estimates the interaction through an empirical equation (eq. 1). This equation is derived from the energies of interactions between a simulated ligand when interacting with a binding site and the same interactions of the free ligand in the solvent:


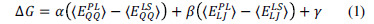


In terms, 
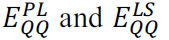
 are the electrostatic interaction energies of the ligand in the complex and free in the solvent; 
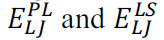
 are van der Waals interactions, and the α, β, and γ derive from a parameterized linear fit to reproduce free energies of known interactions. Therefore, they are arbitrary parameters.

The MM/PBSA [47] method is a state's free energy being estimated from the following sum (eq. 2):


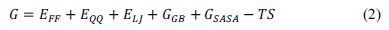


These three terms represent standard force field terms (E_FF_), electrostatic interactions (E_QQ_), and van der Waals (E_LJ_) interactions. The polar and non-polar contribution to the solvation-free energy is *G_pol_* and *G_np_*, respectively. A generalized Born (GB) (MM/GBSA) model can be used to estimate *G_pol,_* and a linear relationship is used to estimate the non-polar term based on the Solvent Accessible Surface Area (SASA). A normal-mode analysis of the vibrational frequencies yields a normal-mode entropy estimate, *S* and *T* as the system temperature. It is usual to simulate only the ligand-protein complex and then subtract the appropriate terms (E_FF_) to obtain the ensemble average of the free receptor and ligand, simplifying the computation of *ΔG*_bind_ [47].

**Fig. (2):**
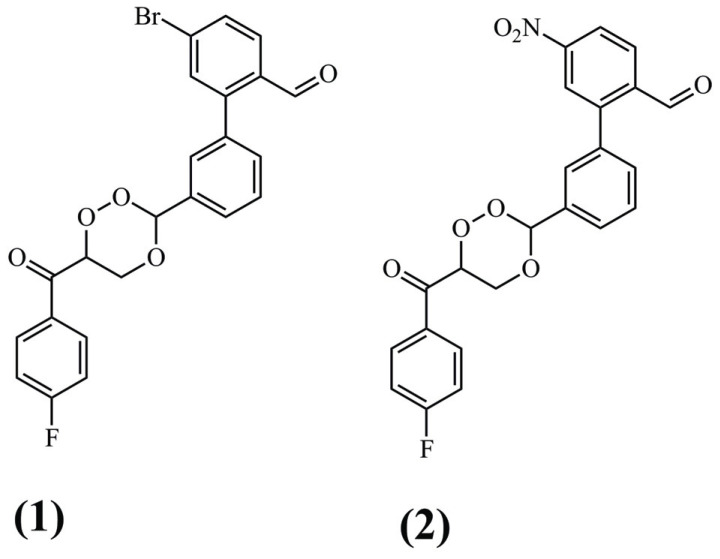
Chemical structure of trioxane analogs (1) and (2).

MM-PBSA successfully verified the affinity of two compounds with the trioxane group, the compound **(1)** (-55,1 kcal mol^-1^) and the compound **(2)** (-62,2 kcal mol^-1^) (Fig. **2**) against *P. falciparum* targeting falcipain in MD simulations of 10 ns, using the Discovery Studio 2020 software [49]. In another study in 2021, this technique was successfully used to determine the affinity of a compound screened from one of the Maybridge databases towards Cathepsin B. The cysteine protease can catalyze the degradation of amyloid plaque precursor protein [50]. The compounds would then have helpful activity in treating Alzheimer's disease. Free-binding energy was estimated using 20 ns simulations performed with the AMBER10 program [50].

### 2.3 Software’s Performance

MD programs have been fueled by their impact on computational research. This simulation emerged as a powerful computational tool, possibly simulating various systems in and out of thermodynamic equilibrium [31]. In recent years, MD software has been used by researchers, among which GROMACS [51], NAMD [52], CHARMM [53], and AMBER [54] stand out, which run efficiently on clusters of computers with distributed memory.

The number of MD tool combinations with graphical interfaces has increased over the years. For example, Martinez and coworkers (2009) [55] developed PACKMOL, a package for building initial setups for MD simulations. This code makes it possible to pack millions of atoms grouped into complex molecules within several 3D regions. The user can only provide the structure of each molecule of each type and the geometric constraints each molecule must satisfy. This way, building complex mixtures, interfaces, solvate biomolecules in water, and other solvents is possible.

Another reported program is RIN-MD, a tool that makes it possible to analyze the residue interaction networks (RIN) in protein molecular dynamics. This program acts as a Visual Molecular Dynamics (VMD) plug-in, facilitating the study of structures. In a RIN analysis, the nodes represent the amino acid residues, their connections, and the non-covalent interactions. In this way, the RINs are generated through the MD trajectory files, including non-covalent bonds, such as H-bond, salt bridges, van der Waals, cation-π, π–π, arginine–arginine and Coulomb interactions, showing the information a 2D interface [56].

Zaczek *et al.* (2019) [57] created the MDMS (Molecular Dynamics Made Simple) program to guide users through the entire process of performing MD. MDMS uses accessible language and flexibility for complex cases, making MD viable for beginners and experts in this field. Initially, it assists in choosing the protein structure, model preparation, parameterization, and simulation execution.

Another program is MDBenchmark, developed by Gecht *et al.* (2020) [58] to speed up the setup, shipping, simulation analysis, and scale study. Developed as open-source software, users can run benchmarks, scale studies, engines, and cluster computing. Streamlining the process and simplifying finding ideal simulation parameters also sends simulations to the queuing system, varying the number of nodes, Central Processing Unit (CPU), and Graphics Processing Units (GPU) usage.

The SINAPs tool was created to analyze and visualize interaction networks from MD simulations in python language, and available free of charge, the program arises to solve the main interactions that distinguish two states of proteins. Additionally, these interactions can be presented in a 3D view through a UCSF Chimera plug-in [59].

Other tools and Web servers have emerged to facilitate structural analysis and provide additional features to the traditionally described methods. ENCoM, a free tool, emerged as a coarse-grained analysis method that considers the nature of amino acids. The study additionally aims to help predict the effect of single-point mutation on protein dynamics and thermostability [60].

CHARMM-gui is a web-based graphical user interface that prepares complex biomolecular systems for MD simulations [61]. Other web servers are MDMoby and MDWeb [62], with automatic configuration functionality, where MDMoby provides all configuration, simulation, and analysis operations. On the other hand, MDWeb is an easy-to-use web-based interface where users can check the input structure's quality and customize their configuration protocols [62]. The PREFMD (Protein REFinement *via* Molecular Dynamics) implements a more extensive MD-based refinement protocol based on the highest-performing refinement method [63].

LARMD was created to perform and analyze MD of up to 4 ns, through a free and easy-to-use online standard protocol, for ligand binding and unbinding analysis [64]. The program calculates Normal Mode Analysis (NMA) from static structures. It also computes Principal Component Analysis (PCA), protein lattice analysis, MM-PBSA, and Dynamic Cross-Correlation (DCC) calculations.

NAPS is a free web server that performs MD simulations and data network analysis. Its applications may indicate analysis of subtle conformational change, flexibility in proteins, and alternative binding pockets for drugs. Its upgrade complements interactive graphics and MD execution on biomolecules such as proteins, protein-protein, protein-nucleic acid complexes, MD pathways, and RNA [65].

DynaMut2 emerged to evaluate changes in the stability and flexibility of protein mutations. This free web server allows it to combine NMA methods, such as LARMD and graph-based signatures, to show protein movement. Furthermore, the program presents the possibility of accurately predicting the effects of mutations on protein stability [66].

The free web server MDM-TASK-web combines other software, such as MD-TASK and MODE-TASK, to perform granular protein analysis. The server allows for performing network analysis of dynamic residuals, perturbation, cross-correlation, and mode analysis. The program aims to investigate the global movement of proteins and intrinsic and extrinsic disturbances, such as aesthetic and orthosteric changes, temperature, pH, and mutations. It also includes metrics such as Residue Interaction Network (RIN) and weighted waste contact maps [67].

Atomevo, developed by Hao *et al.* (2022) [68], is a free web server that integrates a series of easy-to-use tools: *i)* homology modeling of proteins by MODELLER, *ii)* molecular docking by Autodock Vina, *iii)* MD simulation through GROMACS and *iv)* Molecular Mechanics/Poisson-Boltzmann Surface Area (MM/PBSA) analysis. The user can upload input files, configure parameters, and download output files on this server.

In recent years MD has become more accessible. Until recently, most jobs required a supercomputer. However, computer hardware with GPUs was introduced to run simulations at lower costs [69]. The search for an increase in computing power and data processing over the years has been reported by several researchers. These solutions demonstrate possible and applicable options for academia and research. Among the examples, we can highlight the development of new functional forms for interactions [70, 71], new force fields [72, 73], and new integration algorithms [74, 75]. All programs vary in their capabilities and feature set.

### 2.4 Applications in Drug Design and Discovery

**Fig. (3):**
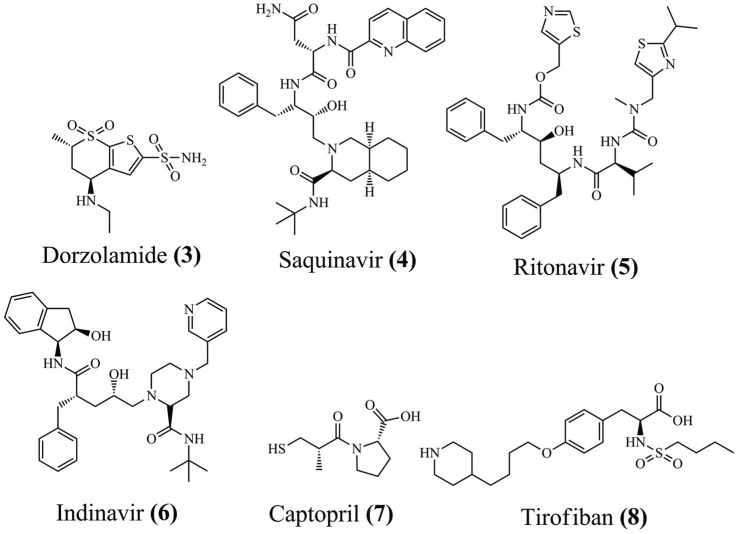
Chemical structure of some drugs discovered using CADD approaches.

CADD methods have seen rapid growth in recent years. Drugs such as dorzolamide **(3)**, saquinavir **(4)**, ritonavir **(5)**, indinavir **(6)**, captopril **(7)**, and tirofiban **(8)** (Fig. **3**) have benefited from using CADD, demonstrating the accuracy of the validated results and their importance in pharmaceutical applications [76]. Molecular docking studies, mainly involving explicit solvents, have supported the characterization of flexible binding sites and the evaluation of binding pathways, kinetics, and thermodynamics. However, limitations related to flexibility instigate researchers worldwide to associate molecular docking with other approaches. Thus, the MD simulations become essential in any drug design campaign [31, 77].

**Fig. (4):**
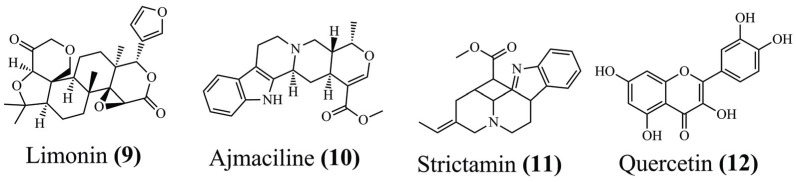
Chemical structure of limonin (9), ajmaciline (10), strictamin (11), and quercetin (12).

MD simulation is becoming increasingly important to identify which molecular properties are essential and the molecular interactions responsible for the binding of ligands in a macromolecule [31, 77]. In this way, the study of Skariyachan *et al.* (2018) highlighted the screening of 104 potential inhibitors against targets of multi-drug-resistant *Acinetobacter baumannii* [78]. Molecular docking results demonstrated strong interactions with nine targets taken from the PDB, and three compounds (Fig. **4**) were selected for MD simulations (limonin **(9)** to diaminopimelate epimerase, ajmaciline **(10)** to aspartate semaldehyde dehydrogenase and strictamin **(11)** to UDP-*N*-acetylglucosamine 1-carboxyvinyltransferase). Thus, MD simulations showed stability up to 250 ns for all compounds, which could be confirmed *in vitro*.

In another study, Lourenço *et al.* (2020) [79] performed virtual screening based on molecular docking to identify the supposed action mechanism of quercetin **(12)** (Fig. **4**) extracted from the plant *Bryophyllum pinnatum (Lam.) Oken*. The authors confirmed the PDE4 (phosphodiesterase-4) enzyme as a promising target by *in vitro* assays, indicating more excellent selectivity for PDE4B than PDE4A. In addition, MD simulations were performed to evaluate the stability of the complex with PDE4B using the five best poses extracted from the docking at a time of 5 ns. The stability of the conformations was presented in the RMSD, while the MM-PBSA highlighted that two of the five poses showed the best energy values (-72.58 kcal mol^-1^ and -72.38 kcal mol^-1^). After extending the simulation time, the two poses demonstrated high stability up to 14 ns, with two π-π interactions and eight H-bonds, identifying the flavonoid derivative quercetin as a promising PDE4B inhibitor.

**Fig. (5):**
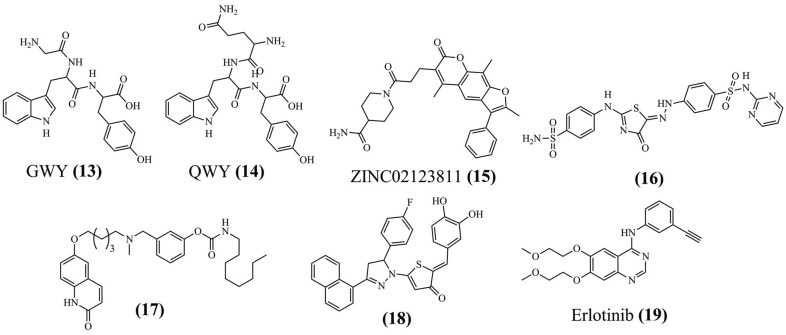
Chemical structure of some compounds identified using MD simulations.

Yan *et al.* (2020) performed an initial screening using 3D-QSAR models, resulting in the identification of two antioxidant tripeptides: GWY **(13)** and QWY **(14)** (Fig. **5**) [80]. Furthermore, molecular docking was applied to identify the potential mechanism resulting in KEAP1. MD simulations confirmed the stability of the compound at 30 ns, being the same site found for NRF2 binding with KEAP1. The RMSD indicated that KEAP1 binding with GWY **(13)** or QWY **(14)** reached equilibrium faster than KEAP1 not binding with the ligand. Regarding energy, the compounds were strongly linked, with electrostatic and van der Waals interactions significant for combination, and R_g_ results showed that GWY **(13)** seemed more stable than QWY **(14)**.

On the other hand, Jairajpuri *et al.* (2021) identified natural compounds as inhibitors of the SARS-CoV-2 M^pro^ [81]. Initially, 90,000 compounds obtained from the ZINC database were analyzed under ADMET and toxicity parameters. Of these, 32,902 compounds were used in molecular docking, which consisted of a filter to identify candidates with the best profile. The most promising compound was selected for MD. The simulation was carried out in two systems: the free protein and the complex with the inhibitor. RMSD demonstrated the stability of the complex, and RMSF showed minimization of fluctuations in the presence of the complex. Thus, initial fluctuation of up to 15 ns in the R_g_ must be due to the packing adjustment of M^pro^, which then remained balanced up to 100 ns. The stability of the complex demonstrated that the compound ZINC02123811 **(15)** (Fig. **5**) presented stable conformations and interactions at 100 ns, maintaining the interaction with the amino acid residue Cys^145^ and His^41^. Thus, computational analysis indicates that this compound can be a scaffold for developing potential inhibitors.

Another computational study was validated *in vitro*, aiming at inhibiting arylhydrazothiazolylsulfonamides analogs for antibacterial and antifungal infections [82]. The best compound **(16)** (Fig. **5**) showed activity against *B. cereus*, *P. aeruginosa*, *E. coli,* and *C. albicans*. The molecular docking and MD simulations were performed with tyrosyl-tRNA synthetase, dihydropteroate synthetase, and *N*-myristoyl transferase to evaluate its possible biological target. MD showed a good binding profile for the simulations, with RMSD and RMSF of <3.5 Å and 1 Å, respectively, over the entire period for the systems. The R_g_ demonstrated that the simulation compaction was similar to the co-crystal ligand. Also, MM-PBSA indicated that the dihydrofolate reductase complex (-144,349 kcal/mol) was the best binding free energy. In this way, the presence of H-bonds that formed the complexes was thermodynamically highly stable.

The existence of native substrates, active sites, and SARs studies of fatty acid amide hydrolase (FAAH) inhibitors and cholinesterases led Maleki *et al.* (2021) to evaluate the action of carbamates as possible inhibitors [83]. Thus, compound **(17)** (Fig. **5**) was evaluated as having the best *in vitro* inhibitory activity against the enzymes. In 100 ns of simulation, MD could determine its form of inhibition in three targets: FAAH, acetylcholinesterase (AChE), and butyrylcholinesterase (BuChe). When comparing with the Apo form of the proteins, the RMSD indicated that the complex with the ligand has more stability. The RMSF shows that the active site was less flexible when linked to compound **(17)** inhibitor. Kinetic studies confirmed the inhibition of compound **(17)**, indicating that it inhibits AChE through the mixed-mode mechanism, and for BuChE, the inhibition mechanism is the non-competitive one.

Computational methods have studied carcinogens due to the wide availability of 3D information in several databases. Thus, Eldehna and colleagues (2022) evaluated the activity of novel 3-(naphthalen-1-yl)-4,5-dihydropyrazoles as EGFR inhibitors as anticancer agents [84]. The inhibitory activity at the nanomolar scale for the most active compound **(18)** (Fig. **5**), with IC_50_ 267 ± 12 nM. Molecular docking was performed to explore the binding mode, highlighting a similar pose related to EGFR inhibitor erlotinib **(19)** (Fig. **5**). The MD simulation of compound **(18)** and erlotinib **(19)** shows the stable RMSD for both, with lower values than the Apo form of the EGFR (1.8, 1.7 and 4.2 Å, respectively). RMSF concluded similarly, with greater flexibility of amino acid residues for the Apo form. The new inhibitor achieved free energy results similar to erlotinib **(19)**, consistent with *in silico* and *in vitro* analysis.

## 3 MD SIMULATIONS TO DISCOVER CYSTEINE PROTEASE INHIBITORS

MD simulations have been widely used to evaluate the internal movement, physical arrangements, and structural changes induced by the environment in proteins and their interactions with other chemical molecules. The MD simulations can describe the dynamics of the binding mechanism of a small molecule to a protein under interference from water, pressure, temperature, and ions [85]. In addition, it is a method constantly explored to discover cysteine protease inhibitors useful against several diseases, mainly parasitic and viral diseases [3, 19]. The following topics will address the main studies using MD simulations to discover cysteine protease inhibitors.

### 3.1 Cruzain (*Trypanosoma cruzi*)

Chagas disease is caused by the protozoan flagellated parasite *Trypanosoma cruzi* and was first described by Carlos Chagas in 1909. The disease was initially endemic to Latin America, but it has spread to other places such as Canada, the United States, Europe, Australia, and Japan, affecting 6-7 million people worldwide, and the number of deaths is approximately 50,000 annually. Despite that, no vaccines are available, and the chemotherapeutic drugs (benznidazole and nifurtimox) are effective only in the acute phase. In addition, 20% of cases must be stopped, providing several side effects necessary to develop the most effective new drugs [86, 87]. In this way, studies focus on the *T. cruzi* enzyme cruzain or cruzipain, a cysteine protease abundant during the parasite's life cycle and are necessary mainly in the amastigote forms [86, 87].

**Fig. (6):**
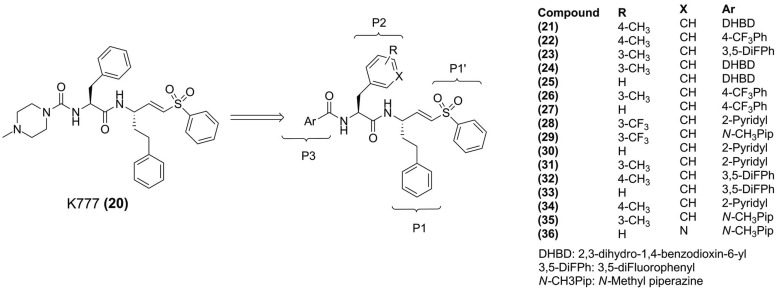
Chemical structures studied by Luchi and collaborators (2019).

To discover helpful information for cruzain inhibitors design, Luchi and collaborators (2019) performed structure-activity relationship (SAR) by applying the quantum theory of atoms in molecules (QTAIM) methodology with ML tools and MD simulations, using known analogs of K777 to investigate the influence of the substituents in P2 and P3 (Fig. **6**) [88]. After analyzing 17 known inhibitors (**21** – **36**) (Fig. **6**), they identified interactions that are prevalent in the most active group (for example, **32**), such as H-bond between side chains of protonated His^162^ and Asn^182^, which favor the thiolate-imidazolium ion pair (Cys^25^-His^162^) necessary for catalysis. In contrast, less active **(34)**, the indole ring of Trp^184^ occupied the space where this interaction was observed, suggesting that Trp^184^ might act as a “switch” for this interaction. MD simulations of the complex with compound **(32)**, the His^162^ was closer to Asn^182^, while in the complex with compound **(34)**, His^162^ was part of the time far away from Asn^182^, confirming the hypothesis proposed by the authors.

**Fig. (7):**
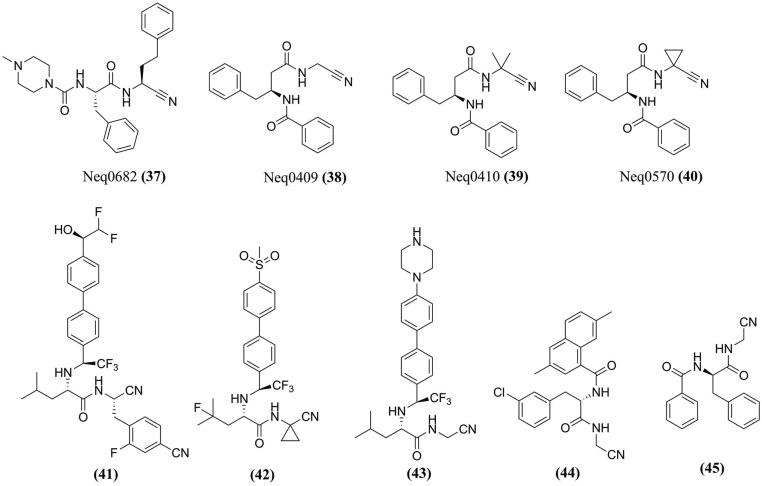
Chemical structure of some cruzain inhibitors studied by MD simulations.

Compound K777 **(20)** (Fig. **6**) is the most successful cruzain inhibitor, with efficacy in acute and chronic Chagas disease models. However, biological studies stopped due to its poor tolerability, possibly because of an irreversible inhibition, which forms an adduct with the sulfur atom of the active site cysteine thiol in cruzain. Therefore, Silva and collaborators (2020) designed a reversible covalent inhibitor based on the formation of thioimidate adduct with the thiol of the catalytic cysteine, which resulted in Neq0682 **(37)** (Fig. **7**) that bears a nitrile, losing the aryl sulfone region of K777 **(20)** and maintaining the same scaffold [89]. The MD simulations showed that the two inhibitors performed the same type of interaction in the active site of cruzain. Finally, the QM/MM shows that Neq0682 **(37)** is a reversible covalent inhibitor, and the reaction free energy of K777 **(20)** is significantly more negative than the reaction for Neq0682. Therefore, the barrier for the reverse reaction is higher for K777 **(20)**, explaining why it is an irreversible inhibitor and providing insights into a covalent inhibition mechanism.

Experimental data suggest that dipeptidyl nitrile inhibitors bind tightly inside the active site of cruzain, and the inhibition occurs through the reversible formation of a covalent bond. Further investigations through MD simulations related to the reaction mechanism of dipeptidyl nitrile derivatives Neq0409 **(38)**, Neq0410 **(39)**, and Neq0570 **(40)** (Fig. **7**) were reported by Santos *et al.* (2018) [90]. The results demonstrated a concerted mechanism where the compounds' proton transfer from His^162^ to N1 occurs with a nucleophilic attack from negatively charged Cys^25^ at C1 of the compounds. Although the binding enthalpy was exothermic for all ligands in the isothermal titration calorimetry (ITC), Neq0570 **(40)** (*Δ*G_bind_= -9.0 kcal/mol) and Neq0409 **(38)** (*Δ*G_bind_= -8.9 kcal/mol) demonstrated better binding affinities than Neq0410 **(39)** (*Δ*G_bind_= -7.5 kcal/mol) and therefore are thermodynamically more favorable.

To evaluate the differentiation capacity of MD simulations among cruzain inhibitors, Sartori *et al.* (2019) [87] selected the dipeptidyl nitriles from the literature, compounds **(41)**, **(42)**, **(43)**, and **(44)** and an inactive, compound **(45)** (Fig. **7**). Concerning **(42)**, the simulations identified a poor geometry for a non-covalent complex with cruzain, even though it is a promising inhibitor of the enzyme. In addition, the complex of **(45)** with cruzain showed excellent stability and geometry. However, the relative orientations of groups are not conducive to nucleophilic attack, which is explained by the fact that this ligand is a poor inhibitor.

**Fig. (8):**
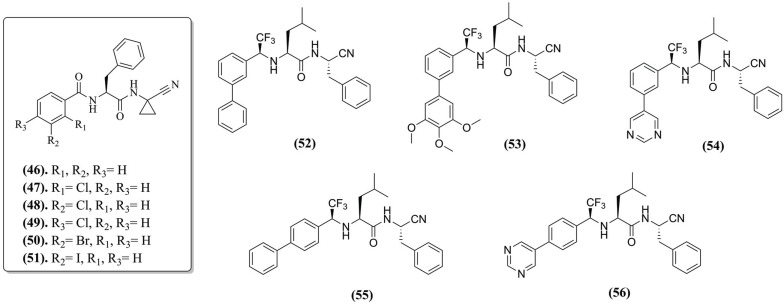
Chemical structure of the compounds developed by Cianni *et al.*

Regarding the covalent nitrile-enzyme complexes, Cianni *et al.* (2018) [91] identified favorable substitutions in dipeptidyl nitriles through three replicates of 100 ns MD simulations with compounds **(46)**, **(47)**, **(48)**, **(49)**, **(50)**, and **(51)** (Fig. **8**). A better interaction pattern was highlighted between the Ser^61^ hydroxyl group and the chlorine atom at the *meta*-position of compound **(48)** compared with *orto*-chlorophenyl in compound **(47)** (most constrained) and *para*-chlorophenyl in compound **(49)** (most flexible). Bromine and iodine at the *meta*-position (compounds **(50)** and **(51)**) also can perform an H-bond interaction with residue Ser^61^ of the cruzain S3 cavity, suggesting the existence of the halogen bond intermediating the bimolecular recognition process.

Continuing the analysis of the influence of P3/S3 interactions by MD simulations, Cianni *et al.* (2020) [92] performed modifications through *meta*-substitution in P3 in the compounds **(52)**, **(53)**, and **(54)** (Fig. **8**) to obtain the potent and selective inhibitors **(55)** and **(56)** (Fig. **7**). Beyond interactions with Gly^66^ and Asp^161^ by H-bonds, residues that dipeptidyl nitrile-like molecules usually bond in the covalent complex. Two different modes of binding (MoB) to the active site were identified: the P3 region of compound **(54)** was in contact with the S3 surface, and CF_3_ was exposed to the solvent (MoB I). CF_3_ interacted with the S3 surface for the other compounds, while the P3 group formed an intramolecular π stacking interaction with the phenyl group at P1 (MoB II) during most of the simulation. The difference is justified by the pyrimidine ring in the *meta* position, which interacts with Ser^61^ by H-bond only during MoB I and do not interact with the same residue when pyrimidine is in *para*-position such as in compound **(56)** and performed the MoB II. However, the difference in pK_i_ values among these compounds is very small, ranging from 8.3 to 9.2 It can be assumed that both MoBs contribute to cruzain inhibition.

**Fig. (9):**
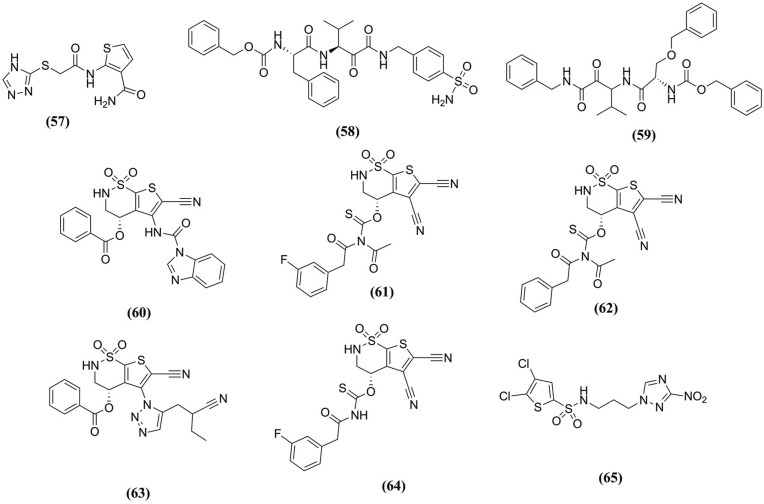
Compounds identified against cruzain using MD simulations.

In another study, Hoelz *et al.* (2015) [93] performed MD simulations to understand the cruzain behavior before and after binding to the inhibitor by using two systems in an aqueous solvent under pH 5.5, one free (the apo form) and the other bound to the Neq 176 **(57)** (Fig. **9**). The analysis showed no significant stability variation during the simulation, so the inhibitor binding does not change the protein fold. According to the C_α_-RMSF plot, only loop-3 (between Cys^56^ and Leu^67^) and loop-4 (between Asp^87^ and Thr^107^) presented structure fluctuations. The binding mode of Neq176 **(57)** occurred only by H-bonds, mainly with Gly^66^, Met^68^, Asn^69^, and Leu^160^ residues. Additionally, the PCA analysis demonstrated that the movements in the apo form system led to an open conformation. However, while the enzyme is bound to the inhibitor, the R_g_ analysis confirmed a closed conformation, where the cruzain apo system showed higher values. Therefore, the inhibitor binding induced conformational changes in the enzyme structure to accommodate the inhibitor.

To identify new cruzain inhibitors based α-Keto scaffolds Saraiva *et al.* [94] selected 31 from the literature, synthesized and tested in the same experimental conditions, aiming to construct 3D-QSAR models. The most active compound **(58)** (Fig. **9**), and the least, compound **(59)** (Fig. **9**) presented pIC_50_= 9.191 and pIC_50_= 7.284, respectively. Thus, MD simulations described that the RMSD value for compound **(58)** is below compound **(59)**. Still, both ligands maintained low deviations, given the presence of strong disulfide bonds that contributed to the stability of the complex. The interactions at the active site were performed mainly with Gln^19^, Cys^25^, Gly^65^, Gly^66^, and Asp^158^ for the least active compound, while the most active one interacted with Asn^175^ because of the sulfonamide group. These hydrophobic groups in compound **(59)** promoted instability, whereas the polar group in compound **(58)**, sulfonamide, induced strong interactions. According to the RMSF plot, the residues Lys^58^, Thr^59^, Asp^60^, and Ser^61^ are responsible for the highest fluctuation (2.45 Å). Finally, the binding free energy of the most active compound **(58)** was favorable (*Δ*G_bind_= -50 kcal/mol) and stable along the simulation, indicating inhibitory interaction.

In another work, Costa *et al.* (2022) applied the *de novo* approach to propose new sulfonamide derivatives with potential action against cruzain based on 146 sulfonamide fragments that were filtered to 5 best compounds **(60)**, **(61)**, **(62)**, **(63)**, and **(64)**, and with compound **(65)** (Fig. **9**) as the reference [95]. Thus, molecular docking suggested that Cys^25^, Gly^65^, Leu^67^, Asn^69^, His^159^, and Ala^133^ played an essential role in molecular recognition. Also, MD simulations revealed the values of RMSD for the compounds **(60)**, **(61)**, **(62)**, and **(65)** were all below 1.50 Å, and in RMSF analysis, some regions, as Trp^7^-Val^34^, Glu^50^-Met^68^, Ala^92^-Ala^110^, Val^135^-Leu^166^, and Ile^194^-Ser^211^ were the most flexible of cruzain. In addition, the R_g_ presented minor variations during the simulation, suggesting the stability of the folded protein. Concerning the MM/GBSA calculations for binding free energy, **(60)** displayed the lowest values among all evaluated compounds, including the reference compound CP6 **(65)**.

**Fig. (10):**
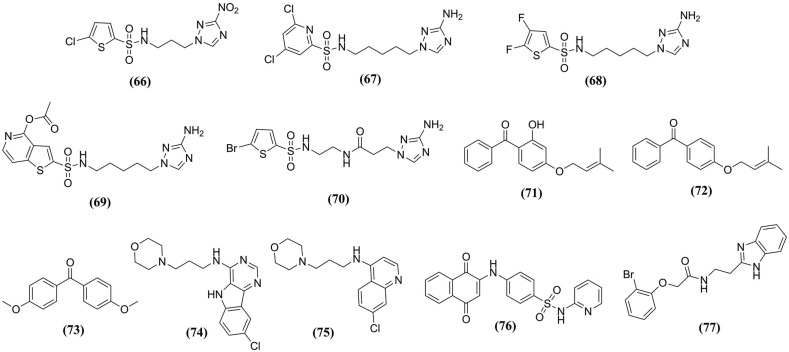
Several chemotypes identified against cruzain studied by MD Simulations.

Souza and collaborators (2021) [96] performed a QSAR study to design new cruzain inhibitors. Thus, were obtained the compounds **(66)**, **(67)**, **(68)**, **(69)**, and **(70)** (Fig. **10**). The compound **(69)** showed high interaction energy and potent biological activity (pIC_50_= 6.93). The MD simulations demonstrated the complex stability, and the loop regions with more significant fluctuations according to RMSF are Val^54^-Leu^67^, Glu^95^-His^106^, and Thr^148^-Gln^159^ (S3 subsite), the binding free energy values determined by MM-GB/PBSA and LIE methods were low (*Δ*G_MM-GBSA_= -29.61 Kcal/mol, *Δ*G_MM-PBSA_= -26.55 Kcal/mol, and *Δ*G_LIE_= -14.48 Kcal/mol). Besides, compound **(69)** interacted with several residues, highlighting Gly^23^, Cys^25^, Trp^26^, Ser^64^, Gly^65^, Gly^66^, Gly^67^, and His^162^, contributing to the fixation and stabilization of the complex.

Additionally, Freitas *et al.* (2018) [97] reported in a previous paper the antileishmanial activity of nine alkyl-substituted benzophenones analogs **(71)**, **(72)**, and **(73)** (Fig. **10**) that evaluated against cruzain. Thus, compounds **(71)** and **(72)** demonstrated potential inhibition with IC_50_ values of 9.51 and 10.86 μg/mL, respectively, and compound **(73)** showed no inhibition. Next, MD simulations were performed, and the RMSD values of the complex with the compounds **(71)** and **(72)** achieved values up to 0.168 and 0.161 nm that indicated downward movement of the enzyme. Similarly, RMSF for complexes with the compounds **(71)** and **(72)** were 0.085 ±0.053 and 0.082 ±0.042, respectively. Additionally, compound **(71)** shows the average value of an H-bond of 2, with 53.5% and 74.1% occupancy for Gln^37^ and Val^214^, while compound **(72)** does not show an H-bond. Finally, the hydroxyl group in the compound **(71)** ring can also be related to the greater interaction affinity, resulting in the lower IC_50_ value for cruzain.

Cruzain is regulated by chagasin, an endogenous inhibitor of papain-like cysteine protease. Furthermore, site-directed mutagenesis analysis in chagasin residues has been executed to elucidate evolutionarily conserved residues' functional role in the inhibition of cruzain. Among them, the mutation in T31 decreased 40-fold the binding affinity for cruzain and, when combined with T32, decreased 140-fold, so these residues are essential for cruzain inhibition. Another mutant, W93A, only impacted cathepsin L. (110-fold lower affinity). In this context, Toman *et al.* (2020) [98] could not relate the lower affinity of chagasin variants T31A and T31A/T32A for cruzain to conformational changes. However, they noticed that the introduction of mutation W93A increased the number of polar interactions with cruzain. The most common was a salt bridge between residue Arg^91^ of mutant W93A and Asp^18^ of cruzain during 95% of the MD simulation. Moreover, three hydrogen bonds appeared more than 10% of the time, involving the following W93A-cruzain residue pairs: Asp^99^ and Gln^21^, His^98^ and Glu^95^, Ala^93^, and Gln^187^.

After combining a high-throughput and virtual screening, Martins *et al.* (2017) [99] discovered compound **(74)** (Fig. **10**), and its optimization provided a series of analogs, highlighting the compound **(75)** (Fig. **10**) with IC_50_= 15 μM. MD simulations demonstrate the quinoline ring was placed at the S2 subsite interacting with Glu^208^, and the morpholinyl group at S3 corroborant with many crystallographic complexes of cruzain inhibitors. The N1 protonated form generated a more stable complex with cruzain than the deprotonated one. According to the predicted pKa and simulation results, it is the experimental binding protonation state of the compound **(75)**. In addition, favored ionization of Glu^208^ carboxyl and propyl-morpholinyl interactions occurred with Cys^25^ and Asp^61^. The RMSF of the clusters showed similar patterns and was not statistically different among them, indicating similar ligand stability.

Exploring MD methods, Silva *et al*. (2021) [15] executed a virtual screening including 120 analogs against Cruzain. In this way, fourteen 1,4-naphthoquinone-based compounds were selected and synthesized, and the compound JN-11 **(76)** (Fig. **10**) was identified as a *hit* with IC_50_= 6.3 μM. Moreover, the results of C_α_ RMSF of MD simulations identified values ranging from 0.1 to 0.15 nm, and stabilization occurred after 15 ns and remained during all simulation time (100 ns). Also, the area accessible to the solvent did not present significant variations. Thus, JN-11 **(76)** did not favor significant conformational changes in primary amino acid residues and remained at the active site. JN-11 **(76)** demonstrated interactions with Leu^67^, Ala^138^, and Leu^160^ (π-alkyl); Cys^25^ and Met^68^ (π-sulfur); Trp^26^, Gly^65^, Gly^66^, and His^159^ (van der Waals), and Gly^23^ and Gly^163^ (H-bonds) residues, which are associated with the inhibition of the target.

Santos *et al.* (2019) [100] developed non-covalent benzimidazole inhibitors, and after SAR and QSAR studies against Cruzain, the binding mode of the *lead* compound **(77)** (Fig. **10**) to cruzain. The X-ray crystallography has been solved to comprehend the benzimidazole ring's contribution, which is essential for enzyme inhibition. MD simulations showed significant changes in the H-bond profile for different protonation states. The ligand protonation in the linker region was more stably bound to the enzyme by H-bonds with Gly^66^ and Asp^161^ backbone atoms, as noticed in crystallographic complexes with cruzain. Moreover, the extra H-atom in the benzimidazole ring formed H-bonds with Asp^161^ and Ser^64^ with occupancy between 45% and 80% during the simulation. The benzimidazole ring was the ligand region with higher flexibility, while the bromophenyl ring and the linker anchored the compound in the binding site.

### 3.2 Rhodesain (*Trypanosoma brucei*)

Human African Trypanosomiasis (HAT), also known as sleeping sickness, is an endemic parasitic disease and affects 36 countries in sub-Saharan Africa, with approximately 10,000 new cases reported yearly. This disease is caused by two subspecies of *Trypanosoma*, which are *Trypanosoma brucei gambiense* and *Trypanosoma brucei rhodesiense,* responsible for the chronic and acute forms of the disease, respectively and the last one possesses a higher mortality rate. The current HAT therapy involves suramin and pentamidine for the hemolymphatic stage, while melarsoprol, eflornithine, and nifurtimox are applied in the neurological stage. More recently, the nitroimidazole derivative fexinidazole was approved by the FDA for both stages. However, the antitrypanosomal agent's available present problems with dosing schedules, toxicity, and drug resistance, revealing the need to develop new effective drugs against novel targets [101-103].

**Fig. (11):**
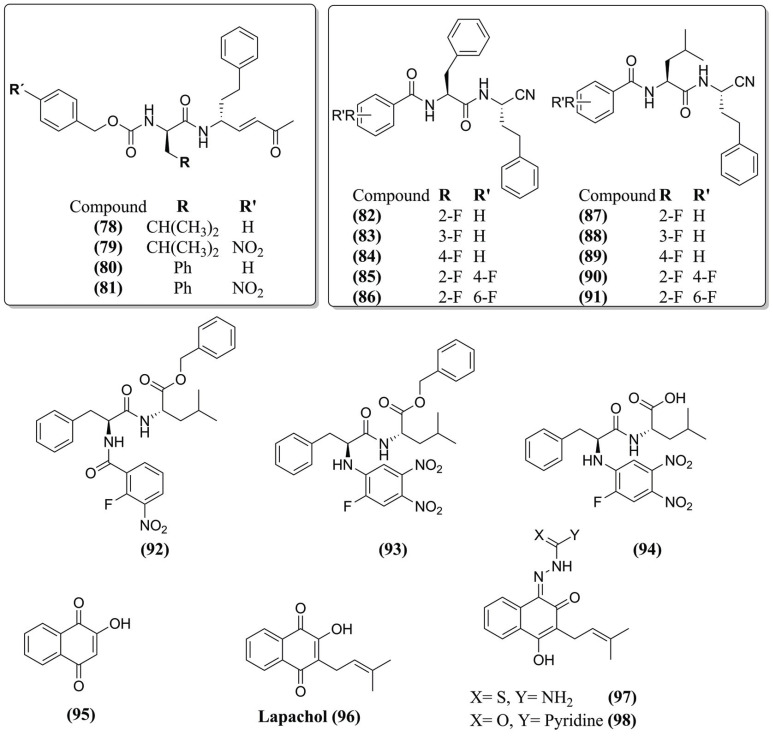
Chemical structure of the compounds rhodesain inhibitors studied by MD simulations.

Therefore, Previti *et al.* (2017) [102] developed peptide-based rhodesain inhibitors with Michael acceptors groups to promote covalent inhibition, identifying the compounds the vinyl ketone analogs **(78)**, **(79)**, **(80)**, and **(81)** (Fig. **11**) as the most promising (K_i_ values ranging from 0.038 nM to 0.9 nM). Further studies against cultured *T. b. brucei* showed the most active compounds **(79)** and **(80)** with IC_50_ of 2.48 and 2.97 μM, respectively. In addition, parasites treated with compound **(80)** exhibited growth retardation with an EC_50_ value of 3 μM. Molecular docking of compound **(80)** showed H-bonds with Gly^66^ and Asp^161^ and accepted two H-bonds from Trp^184^. For the P2 region, the studies confirmed that bulky Phe residue fitted better into the hydrophobic S2 binding site, including Ala^208^, Leu^160^, Ala^138^, Met^68^, and Leu^67^ residues, which justifies the increased activity of the Phe-containing derivatives. The covalent complex with compound **(80)** was analyzed by MD simulation at 80 ns, and out of the 5 H-bonds 4 were maintained for more than 50% of the time, supporting the predicted contacts.

Chio *et al.* (2022) [101] performed a preliminary screening at 10 μM, and all compounds (**82-91**) (Fig. **11**) were revealed to be reversible inhibitors with K_i_ values ranging from 16 to 122 nM, with compound **(83)** better than the standard (K_i_= 35 nM). Next, molecular docking of compound **(83)** covalently docked formed a series of H-bonds with close residues (Gly^66^ and Asp^161^). In MD simulations, the RMSD stability during the simulation did not deviate more than 3.2 Å during 100 ns. However, in the last 20 ns, a slightly different conformation was observed closer to the S1’ subpocket (Gln^19^, Met^145^, His^162^, and Trp^184^). It formed charge-transfer interactions with the aromatic residues of His^162^ and Trp^184^, and the thioimidate NH demonstrated a preference towards Trp^184^ instead of Asp^161^ for H-bonding. Regarding the fluctuations, it is noted that most of the ligand is stable, and the P1 position-hPhe is flexible due to the solvent exposure and the shift mentioned previously. In addition, the P3 phenyl ring and the fluorine substituent in the *meta* position presented moderate fluctuation.

In contrast to classical electrophilic groups such as Michael-acceptor systems, there are (hetero)aromatic electrophiles that react through nucleophilic addition or substitution reactions. In this context, Klein *et al.* (2020) [104] used these groups to design new rhodesain inhibitors. Thus, in a screening identified, the compounds **(92)** and **(93)** (Fig. **11**) were pointed out as the most active (75 and 45%, respectively). In addition, compound **(93)** was hydrolyzed, yielding the free acid compound **(94)** (Fig. **11**) (K_i_ of 4 nM). Molecular docking for compound **(93)** shows multiple poses closer to the cysteine (3.2 Å), suitable for nucleophilic attack initiating the ester hydrolysis. The inhibitor sometimes left the enzyme in MD simulations, but the enzyme-inhibitor complex never split completely. For compound **(94),** the electrophilic aromatic ring distance to cysteine was low (2.7 Å) compared to compound **(93)**. MD simulations indicated a very stable complex and more stable conformation between the sulfur center of Cys^25^ and the proton of the NH substituent occurred when the distance decreased around 2.2 Å, indicating a strong H-bond. Finally, compounds **(93)** and **(94)** presented EC_50_ values of 0.0953 and 18.5 μM in the *T. b. brucei* cell survival assay. These differences are related to the hydrophilicity of the acid **(94)**, which resulted in different cell permeabilities of the compounds.

In previous studies, Santos *et al.* (2019) [100] showed that a novel class of benzimidazole inhibitors presented activity against rhodesain at nanomolar and had trypanocidal activity, which led to a SAR for this class against the enzyme. MD simulations were performed and reveled the protonated and neutral states were analyzed due to the coexistence of the two possible states at the assay pH value of 5.5 using the compound **(77)** (Fig. **10**). The protonation of compound **(77)**, its linker region, was more stably bound to the enzyme due to the formation of H-bonds with Gly^66^ and Asp^161^ backbone atoms. Also, it was observed that the extra hydrogen atom in the benzimidazole scaffold could bond with the side chains of Asp^161^ and Gly^64^ with occupancy between 45-80% of the simulation time, while in the neutral state, the benzimidazole nitrogen hydrogen formed bonds mostly with water molecules.

Due to the potential of 1,4-naphthoquinone-based compounds demonstrated trypanocidal properties, Silva *et al.* (2021) [15] executed a virtual screening of a small in-house library of 120 natural and nature-based compounds against rhodesain and selected fourteen compounds potentially active. Thus, 2-OH-NPQ **(95)**, lapachol **(96)**, AS12/15 **(97)**, IK-01 **(98)** (Fig. **11**), and JN-11 **(76)** (Fig. **10**) were the most active compounds (IC_50_ values of 33, 58, 28, 20 and 1.8 μM, respectively). MD simulation on rhodesain complexed with the compound **(76)** shows complex stabilization after 15 ns, remaining stable during all the simulation time (100 ns). RMSF plot revealed low fluctuations for the residues and even minor fluctuations for the catalytic triad. R_g_ plot presented conformational changes ranging from 1.62 to 1.63 nm, suggesting high rigidity and compactness. The SASA analysis did not show significant modifications varying from 93 to 103 nm^2^, confirming that JN-11 **(76)** does not change the protein structure and remains at the active site.

### 3.3 Falcipain (*Plasmodium spp.*)

Malaria is one of the most prevalent diseases in the world. Despite intense efforts to fight the disease, hundreds of people are infected, and approximately 1 to 2 million deaths occur yearly [105]. *Plasmodium falciparum* is the most lethal malaria parasite among all other Plasmodium strains. These parasites are unable to biosynthesize some essential amino acids. Therefore, parasite survival ultimately depends on generating free amino acids by degrading hemoglobins in the host's erythrocytes. Several proteases are involved in this degradation cascade, such as falcipains, a group of cysteine proteases similar to papain, which are well characterized. Among falcipains, falcipain-2 (FP-2) is the most crucial protease in this cascade. It is overexpressed during the erythrocyte stage of the parasite. It cleaves the skeletal proteins of the erythrocyte membrane in the late stages of parasite development, causing membrane instability, which facilitates the release of the parasite *in vivo* [105]. So far, several antimalarials, for example, quinine, chloroquine, artemisinin, and atovaquone, have been discovered. However, resistance to available drugs is becoming a significant health problem, and it becomes necessary to discover new antimalarials [106].

**Fig. (12):**
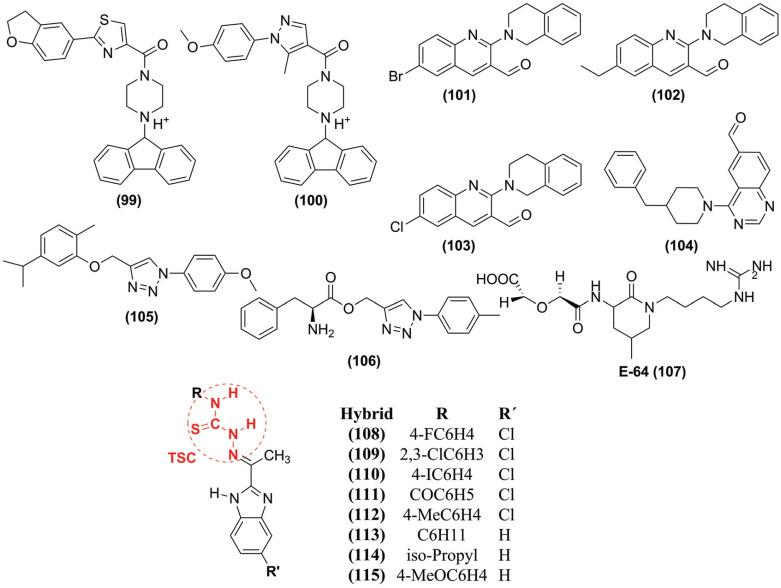
Chemical compounds studied by MD simulations against FP-2.

Two cysteine C1, FP-2, and falcipain-3 (FP-3) proteases were identified as promising drug targets among the currently known *P. falciparum* hemoglobinases. Thus, Hernández-González *et al.* (2018) explored MD simulations using the compounds **(99)** and **(100)** (Fig. **12**) against FP-2 in a 100 ns trajectory [106]. The condensed aromatic rings of both compounds occupy the S2' portion of the enzyme. They probably establish stacking interactions π-π with the residue Trp^206^, as described for other complexes involving cysteine C1 proteases. In addition, the fractions 2,3-dihydrobenzofuran-5-yl and *p*-methoxyphenyl of compounds **(99)** and **(100)**, respectively, are placed in the subsite S2, the primary determinant of FP-2 specificity. This observation agrees with the well-known preference of FP-2 for hydrophobic (aliphatic and aromatic) amino acid side chains in the P2 position. The carbonyl groups occupy subsite S1, according to the already established propensity of electrophilic groups to interact with the catalytic residue (Cys^42^).

Another study, performed by Rajguru *et al.* (2022) [107], evaluated the stability and flexibility of FP-2 complexes with PubChem compounds **(101)**, **(102)**, **(103)**, and **(104)** (Fig. **12**) by MD simulations at 20 ns. In this way, the RMSD shows that all systems are stable during the simulation. The mean value of RMSD varies from 2 to 3.5 Å for the protein-ligand complexes. However, compound **(104)** showed lower stability than the other complexes. The compound **(101)** showed a comparable RMSD with the standard compound, suggesting it is more promising than the other three compounds analyzed. The RMSF showed that the amino acid residues fluctuations were higher around the residues 50-60 and 150-250. The R_g_ for the **(101)**, **(102)**, and **(103)** were more stable than compound **(104)**. Also, compound **(101)** shows more H-bond around the trajectory. However, H-bonds were interrupted at three-time intervals, around 7500 ps, 12500 ps, and 15000 ps. Thus, H-bond analysis suggested a better binding mode between the receptor and the promising compounds, as seen with the standard compound.

Uddin *et al.* (2020) [85] conducted MD simulations lasting 30 ns to evaluate the conformational changes, stability, and interaction mechanism of the selected compounds **(105)** and **(106)** (Fig. **12**). In the RMSD, the mean deviation was up to 10 ns, possibly due to initial orientation in the falcipain-2 (FP-2) binding site. Subsequently, in FP-2, the ligands showed equilibrium along the simulation trajectory, suggesting sufficient stability of the protein-ligand complexes. The ligands achieved a larger R_g_ than the free FP-2, which balances during the simulations, with a lower structural deviation after bonding. The RMSD, RMSF, and R_g_ showed that the FP-2-JMI-105 complex was more compact and stable. The H-bond plots show an average of three for both around the simulations. It was observed that compound **(105)** and compound **(106)** bind at the FP-2 binding site with 5 to 6 conventional hydrogen bonds. Finally, the authors suggested that the compound **(105)** can be a potential leader against FP-2 and can be evaluated for developing drugs against malaria.

Through the studies of Salawu (2018) [108], it is possible to analyze the atomic details of how E-64 **(107)** (Fig. **12**) binds to FP-2. Thus, the studies show that E-64 **(107)** interacting with Asp^170^, Gln^171^, Cys^168^, Gly^169^, Ala^151^, and Gly^230^ (recruiter group A - RA) or interacting with Lys^76^, Asn^77^, and Asn^81^ (recruiter group B - RB). The results show that, in most cases, E-64 **(107)** does not bind directly/immediately to the active site of FP-2 but approaches FP-2 by interacting first with the residues in RA and RB at about 80% and 14% of the time, respectively. On average, the movement of E-64 **(107)** to the binding site reached equilibrium and stabilized around 55 ns. From the results of MD simulations with adaptive polarization (ABMD), the Gibbs free energy is approximately −12,2 ± 1,1 kJ/mol based on the three sets of reaction coordinates/collective variables used. The ABMD simulations reveal interaction favorably with Asn^173^, Asp^170^, His^174^, Ser^149^, Ser^205^, Lys^172^, Asn^38^, Asn^81^, and Asn^86^, comparable to those of the X-ray structure (with RMSD of 3.19 Å, 3.08 Å, and 2.90 Å).

Nkungli *et al.* (2022) [109] investigate hybrid benzimidazole-tiosemicarbazone (**108** – **115**) (Fig. **12**) as potential inhibitors of falcipain-2 (FP-2). Thus, compound **(109)** exhibits the lowest mean free binding energy (-30.32 kcal/mol) calculated by MM/PBSA, and compounds **(111)** and **(112)** showed less affinity. In addition, complex FP-2 with compound **(109)** led the most stable RMSD between 55 and 150 ns of simulation, and no sharp fluctuation was observed during the simulation. Interestingly, the mean RMSD of the protein in the **(109)** complex remained below 3 Å, indicating the stability of the complex is strongly bound to the protein without significantly disturbing its secondary structure. Also, RMSF shows fluctuating residues within the 185-195 range, corresponding to a region randomly wrapped protein loop. Next, R_g_ was between 1.80 - 1.87 nm, which implies that the binding of compound **(109)** to FP-2 does not induce any noticeable structural change. Finally, the H-bonds play a crucial role in determining the binding strength of the protein-ligand.

### 3.4 M^pro^ (SARS-CoV-2)

Given the crucial role of SARS-CoV-2 in viral replication, inhibition of SARS-CoV-2 M^pro^ is considered an attractive target for addressing small molecule oral antiviral therapies to treat COVID-19 [110]. Its potential as a drug target is due to the characteristic cleavage of peptide sequences after the glutamine residue, in which no human protease has this function. This makes it an excellent drug target [5, 14, 19, 111-113].

**Fig. (13):**
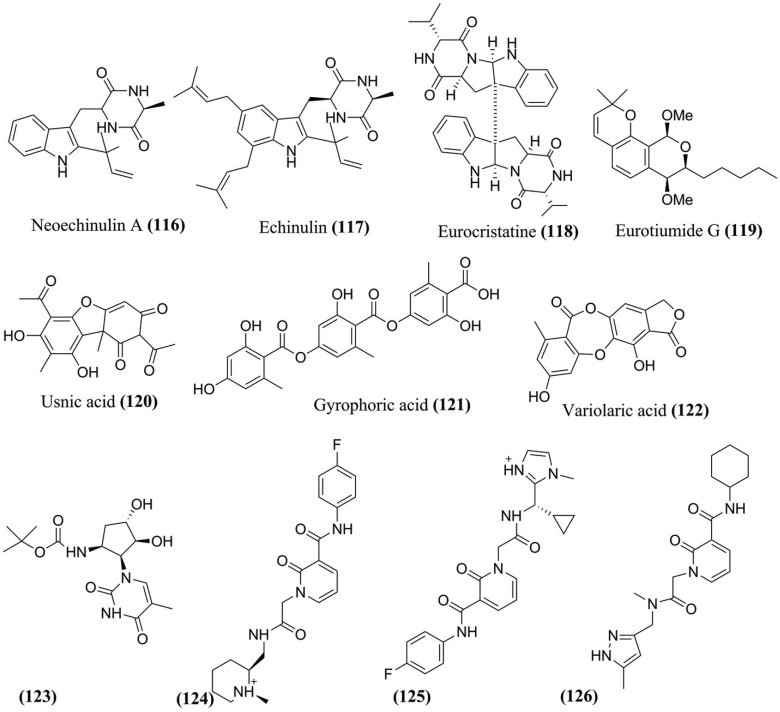
Main compounds identified and evaluated using MD simulations targeting M^pro^ from SARS-CoV-2.

In this context, Alhadrami *et al.* (2022) [114] screened 100 extracts, including 20 marine and 15 terrestrial fungi extracts cultivated in different culture media against SARS-CoV-2 M^pro^, and identified *Aspergillus fumigatus* extract derived from the Red Sea as potential. Studies have led to the identification of the isolated metabolites as two Dicetopiperazines indol prenylated (DKP), neoechinulin A **(116)** and equinulin **(117)**; an indole dimer DKP eurocrystalline **(118);** and an isocoumarin derived from eurotiumide G **(119)** (Fig. **13**). Next, MD simulations of Eurocrystalline **(118)** was the least stable structure within the M^pro^, with an initial binding pose, and achieved RMSD > 7 Å at the end of MD. Neoequinulin A **(116)** and equinulin **(117)** achieved stability, with mean RMSDs of 2.16 Å and 2.21 Å, respectively. The neoequinulin A **(116)** and equinulin **(117)** bonding poses revealed stable H-bonds with Leu^141^, Asn^142^, Gly^143^, and Glu^166^ and significant hydrophobic interaction with His^41^.

Gupta *et al.* (2022) [114] performed the MD simulation at 100 ns to understand the stability of the protein-ligand complexes of usnic acid **(120)**, gyrophoric acid **(121)**, variolaric acid **(122)**, identified against M^pro^ (Fig. **13**). Thus, the RMSD values for usnic acid **(120)** (0.1 to 0.15 nm), gyrophoric acid **(121)** (0.12 to 0.26 nm), and variolaric acid **(122)** (0 to 0.1 nm) indicating stability at the active site. In addition, the RMSF (0.1 to 1 nm) indicates the stability of the complexes. The M^pro^-gyrophoric acid **(121)** complex presented more significant fluctuation in the number of H-bonds (6 to 4), and minimal fluctuation in the number of hydrogen bonds was observed in the M^pro^-usnic acid **(120)** complex (0 to 2) during the 100 ns simulation. The usnic acid **(120)** and variolaric acid **(122)** complexes seem to have lower RMSD values, minimum fluctuation in the RMSF values, adequate H-bonds, and low R_g_, indicating they were forming a highly stable complex.

Mohan *et al.* (2021) [115] screened 8.722 antiviral compounds from the ASINEX library against SARS-CoV-2 M^pro^. The Glide score was used and selected as the four most promising (Fig. **13**). Next, MD simulations with the compounds **(123)**, **(124)**, **(125)**, and **(126)** (Fig. **13**) show the most excellent values of RMSD for the complexes (6.1 Å, 6.3 Å, 10.8 Å, and 10.6 Å, respectively), and RMSD values of the ligand (4.7 Å, 6.3 Å, 8.8 Å, and 5.9 Å, respectively). The compounds showed RMSF 2.7, 1.9, 1.6, and 4.8 Å, respectively. The H-bond plots revealed the compound **(123)** interacted with Thr^98^, Gln^189^, and Asn^142^. On the other hand, compound **(124)** shows interactions with Cys^145^, Thr^26^, Glu^166^, and Pro^168^, and compound **(125)** with the residues Phe^8^, Ser^10^, Asn^142^, and Glu^166^. Finally, compound **(126)** interacted with Gln^189^, Ser^10^, and Asn^142^. All these interactions resulted in increased stability of the protein-ligand complex.

**Fig. (14):**
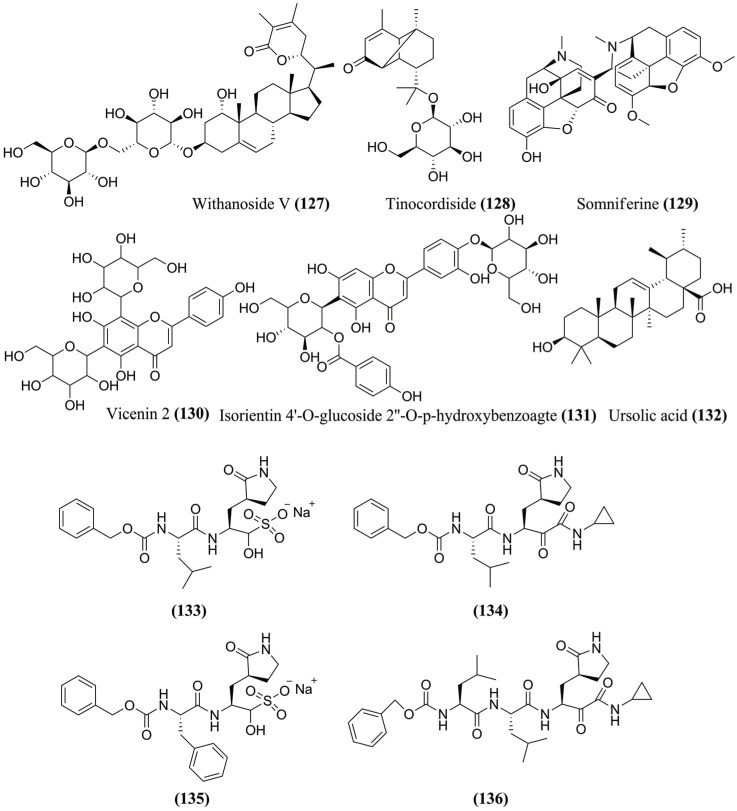
Other compounds identified against M^pro^ from SARS-CoV-2 using MD simulations.

Shreea *et al.* (2020) [116] performed MD simulations of six compounds (**127** – **132**) (Fig. **14**) against M^pro^ to understand the structural deviations in 20 ns of simulation. Thus, the compounds show RMSD acceptable. Except for tinocordiside **(128)** and vicenin **(130)** (Fig. **14**), at initial 8 ns, all other compounds showed minor and stable deviations from 1 to 2.5 Å. Tinocordiside **(128)** and vicenin **(130)** compounds showed variations of up to 3 Å, mainly from 2 to 4 ns of the MD simulations. Ursolic acid **(132)** shows great activity with the binding of loop structures at the M^pro^ binding site, showing 3 Å to 4 Å of RMSD between the 10-20 ns time scale of the MD simulations. Tinocordiside **(128)** offers H-bonds with Arg^188^ and Gln^189^. It was noted that the similarity of H-bonds decreases after 8 ns, impacting the upward movement of the RMSD from 9 to 20 ns. The ligand Isorientin 4'-O-glucoside 2”-O-p-hydroxybenzoate **(131)** (Fig. **14**) showed 8-12 H-bonds up to 10 ns. This impact was revealed in RMSD values, but interestingly, one of the amino acid residues, Glu^166^, was playing a vital role in maintaining Isorientin 4'-O-glucoside 2”-O-p-hydroxybenzoate **(131)** throughout the MD simulations. During the MD simulations, the Withanoside V **(127)** (Fig. **14**) resulted in high residual fluctuations between the regions of the 130-190 position. The amino acid residue Glu^166^ was also vital in maintaining Withanoside V **(127)**, although large deviations were observed in the positions of the residual amino acids.

Sacco *et al.* (2020) [117] evidenced that most M^pro^ inhibitors developed contain 2-pyrrolidone or 2-piperidinone at site P1 as a mimetic of glutamine residue. Their studies performed MD simulations on specific M^pro^ inhibitors, such as compound **(133)** and their analogs **(134)**, **(135)**, and **(136)** (Fig. **14**). Previous studies showed that SARS-CoV-2 M^pro^ cleaves polyproteins in P2-P1, where P1' is a residue with a small side chain (Ala, Ser or Gly), P1 is glutamine and P2 is a large, hydrophobic residue such as leucine or phenylalanine. Therefore, the pyrrolidone of the compound **(134)** occupies the subsite S1 leading to additional stabilizing H-bonds. In addition, the small cyclopropyl group in **(134)** fits in the subsite S1', avoiding steric repulsions with the subsite S1' amino acids. The benzyl group P2 in **(135)** fits in the subsite S2 resulting in van der Waals interactions with Met^49^, His^41^, and Met^165^. The MD simulations verified the stability of the interactions within the bond cavity M^pro^. The complexes formed are stable, and the positions of the ligands did not deviate significantly from crystallographic, with values of Cα RMSD less than 2.4 Å and a general ligand RMSD less than 3.5 Å.

### 4 CHALLENGERS AND OPPORTUNITIES IN MD SIMULATIONS TO DESIGN CYSTEINE PROTEASE INHIBITORS

Although MD simulations are at the top of the techniques currently used for identifying potential inhibitors and predicting mechanisms related to catalysis, several challenges remain to be overcome [118, 119]. Among them, the process's high computational and financial cost stands out [120, 121]. On the other hand, GPUs accelerate the speed in obtaining results, the equipment may not always be affordable, and speed is still a limiting factor if the objective is to identify inhibitors on a large scale (high throughput screening - HTS) [19, 120].

In addition, some studies highlight shorter simulations (between 10 and 50 ns) to identify active compounds. In general, ligands that require more adjustments to find their ideal conformation at the binding site leave the site in more extended simulations. Thus, during the simulation, the ligands change their interactions to adapt to potential energies calculated by force fields based on force field physics, in which van der Waals interactions and electrostatic energies unfavorable result in complex separation. Thus, shorter simulations can more accurately assess the stability of the complex. This can help in more accurate validation of compounds identified by molecular docking at greater speed and scale up at high throughput. Therefore, standardizing this protocol style can considerably improve the speed with which hits and leads are identified [120].

Another considerable problem is the accuracy of existing force fields. MD simulations unveil numerous biological mechanisms at the atomic and molecular level, including protein folding, protein-protein or protein-DNA/RNA interactions, membrane proteins, drug transport, and interactions between lipids. However, parameters related to the fields of strength are still limited. This includes amino acid folding, carbohydrates (extended and less defined structures than proteins), and single-stranded nucleic acids. Furthermore, force fields are limited in eventful cellular environments, as interactions involve multiple factors in real-time. Thus, enhancing force fields should provide new opportunities to study these environments in complex biosystems containing various cellular conditions [122, 123].

Further, another problem is the inability to simulate electronic properties to identify catalytic mechanisms involved in cysteine proteases. Despite the growth of hybrid methods, such as the QM-MM that treat the active site as quantum mechanics (QM) through techniques based on density functional theory (DFT) and the rest of the protein as molecular mechanics (MM), there are deficiencies in software that are easy to instrumentation and computational power and simulation time are still high to use this approach. The next challenge is developing more straightforward software to explore this approach faster for high-throughput applications to increase the speed of *in silico* identification of promising molecules [19].

In fact, cysteine proteases constitute excellent drug targets and are constantly explored as targets against the most differentiated types of diseases. However, one of the significant limitations is selectivity. In many cases, cysteine protease inhibitors fail in clinical trials (ex. K11777) due to high reactivity with cysteine proteases present in the human body, which produces side effects that make their development unfeasible. Thus, more and more advances in medicinal chemistry strategies are needed, especially using *in silico* methods such as MD simulations, to identify increasingly high standards of target selectivity and thus enable the clinical development of inhibitors of these targets [3, 7, 19].

## CONCLUSION AND FUTURE OUTLOOKS

CPs are essential for maintaining the normal physiology of many microorganisms and are considered excellent drug targets. In recent years, their potential as drug targets has become clear, mainly in the search for new drugs against Chagas disease, sleeping sickness, Malaria, and COVID-19. Drug developers increasingly seek higher standards of target selectivity to avoid off-target reactivity, resulting in more selective inhibitors with fewer side effects. In this way, the potential of molecular dynamics simulations emerges, with the function of unlocking aspects involved in the catalysis of these targets that help design promising inhibitors. Despite advances in software development, high-performance computing, and improved force fields, challenges remain for MD simulations to provide an increasingly accurate simulated biological environment for high-throughput screening applications. In this way, it accelerates the speed at which *leads* and *hits* are discovered and increases the probability of developing molecules against various pathogens that threaten the health of the world's population.

## LIST OF ABBREVIATIONS

CPs: Cysteine Proteases
DCC: Dynamic Cross-Correlation
MD: Molecular Dynamics
MDMS: Molecular Dynamics Made Simple
NMA: Normal Mode Analysis
PCA: Principal Component Analysis
PREFMD: Protein Refinement *via* Molecular Dynamics
RIN: Residue Interaction Networks
RMSF: Root-Mean-Square Fluctuation
VMD: Visual Molecular Dynamics
